# Osteochondroma of ventral scapula associated with chest pain due to rib cage compression: A case report

**DOI:** 10.1097/MD.0000000000010510

**Published:** 2018-04-27

**Authors:** Dong-il Chun, Jae-ho Cho, In Ho Choi, Young Yi, Jun Yong Kim, Jae Heon Kim, Sung Hun Won

**Affiliations:** a Department of Orthopaedic Surgery, Soonchunhyang University Hospital Seoul, Daesagwan-ro, Yongsan-gu, Seoul, Korea; b Department of Orthopaedic Surgery, Chuncheon Sacred Heart Hospital, Hallym University, Sakju-ro, Chuncheon-si, Gangwon-do; c Department of Pathology, Soonchunhyang University Hospital Seoul, 59, Daesagwan-ro, Yongsan-gu; d Department of Orthopaedic Surgery, Seoul Foot and Ankle Center, Inje University, Jeo-dong, Jung-gu; e Department of Urology, Soonchunhyang University Hospital Seoul, Daesagwan-ro, Yongsan-gu, Seoul, Korea.

**Keywords:** chest wall, compression, osteochondroma, ventral scapula

## Abstract

**Rationale:**

The scapula is relatively rare site for osteochondroma. Scapula osteochondroma is usually asymptomatic, however it may present with features such as pseudowinging, snapping scapula, bursa formation, chronic pain, and cosmetic deformities. To our best knowledge, this is the first report in the English literature about osteochondroma of ventral scapula associated with chest pain due to rib cage compression.

**Patient concerns:**

A 14-year-old boy was transferred to the orthopedic clinic from thoracic surgery department with a complaint of intermittent, dull, and diffuse aching pain around left chest wall and back from the past 2 months. The patient was previously diagnosed with multiple osteochondromas on another side; proximal tibia and distal femur.

**Diagnosis:**

A radiopaque mass like lesion was observed on the scapula in the posteroanterior view of the chest, and compression of chest wall was also seen. In chest computed tomography (CT), pedunculated outgrowing bony mass was noted in the anterior aspect of the left scapular wing, which showed the continuity of bony cortex and medulla. This bony mass showed the mass effect on the left chest wall, causing left thoracic cavity deformity by making it narrower than the right

**Interventions:**

Surgery was performed under general anesthesia. After the surgery, the arm was immobilized by putting it in an abductor pillow brace for 3 weeks, and during that period pendulum exercise was permitted.

**Outcomes:**

The patient's symptoms resolved in the immediate postoperative period. At 1 year's follow-up, the patient was symptom free and there was no evidence of recurrence of the tumor.

**Lessons:**

We recommend that in case of patients who have a history of osteochondroma and complaint of chest pain, surgeons should become suspicious of the presence of osteochondroma of the ventral scapula. In this situation, we recommend chest radiography, pulmonary function test, and chest CT for early detection and treatment.

## 1 Introduction

Osteochondroma is a benign tumor of the bone and cartilage of unknown etiology.^[1]^ The most common site osteochondroma is the metaphysis of tubular long bones, with the distal femur, proximal tibia, and proximal humerus constituting 90% of the occurrence site.^[1]^ Flat bones such as the pelvis and the scapula are relatively rare sites for osteochondroma with a 3% to 4.5% involvement of the scapula.^[2]^ Usually, osteochondroma is asymptomatic; however, it may present with features such as pseudowinging, snapping scapula, bursa formation, chronic pain, and cosmetic deformities.^[3–5]^ A few case reports have presented such symptoms of osteochondroma of the ventral scapula. However, there exists no report of osteochondroma of ventral scapula associated with chest pain due to rib cage compression. Here, we present a case with a complaint of chest pain due to rib compression of osteochondroma of the ventral scapula.

## 2 Case description

This case report was approved by the Institutional Review Board of Soonchunhyang University Hospital, and informed consent was received by patient.

**Figure 1:**
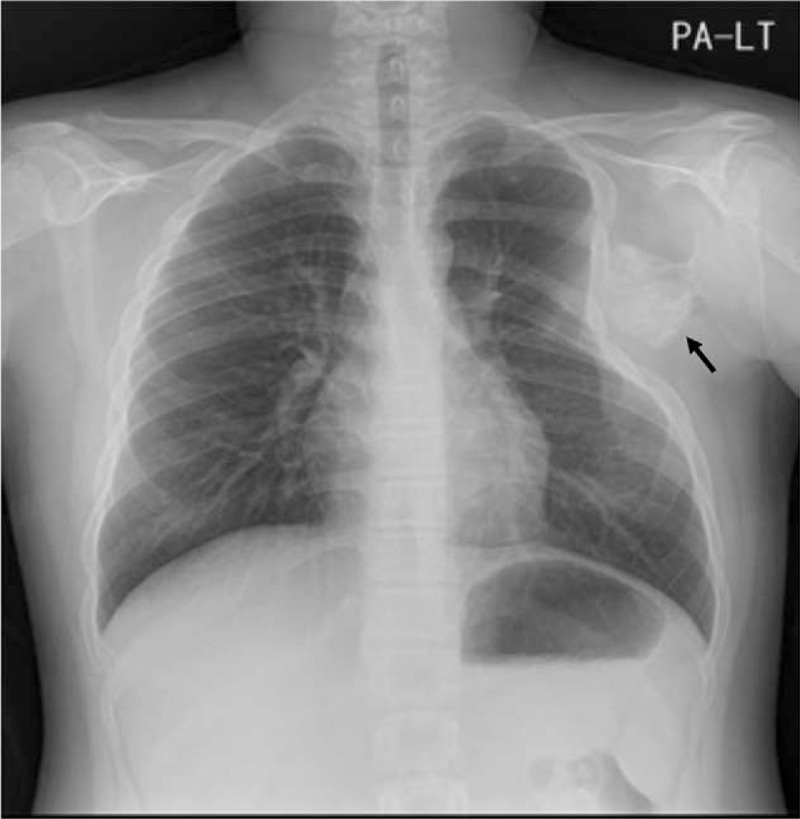
A huge radiopaque lesion is observed on the scapula in the chest anteroposterior view, and compression of the chest wall is seen.

A 14-year-old male was transferred to the orthopedic clinic from thoracic surgery department with a complaint of intermittent, dull, and diffuse aching pain around left chest wall and back from the past 2 months. First, the patient visited thoracic surgeon with these symptoms. There was no marked gross deformity, and all neuromuscular examinations were normal. A radiopaque mass like lesion was observed on the scapula in the posteroanterior view of the chest, and compression of chest wall was also seen (Fig. 1). The patient was previously diagnosed with multiple osteochondromas on another side such as proximal tibia and distal femur.

**Figure 2:**
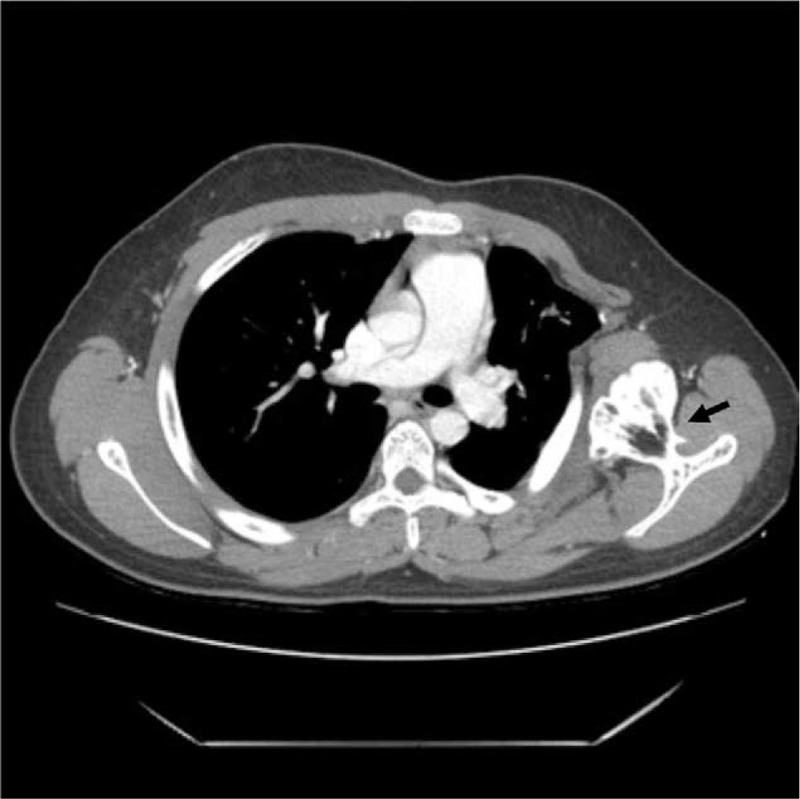
In chest CT, about 5.0 × 4.6 × 5.7 cm sized pedunculated bony lesion is noted in the ventral aspect of left scapula, which shows the continuity of bony cortex and medulla.

We suspected osteochondroma in the scapula and performed chest computed tomography (CT) and pulmonary function test for preoperative examination. In chest CT, pedunculated outgrowing bony mass was noted in the anterior aspect of the left scapular wing, which showed the continuity of bony cortex and medulla. This bony mass showed the mass effect on the left chest wall, causing left thoracic cavity deformity by making it narrower than the right (Fig. 2). Pulmonary function test showed normal pulmonary function with no abnormalities in lung volume or respiratory function. We decided to perform surgery to excise the lesion on the scapula.

**Figure 3:**
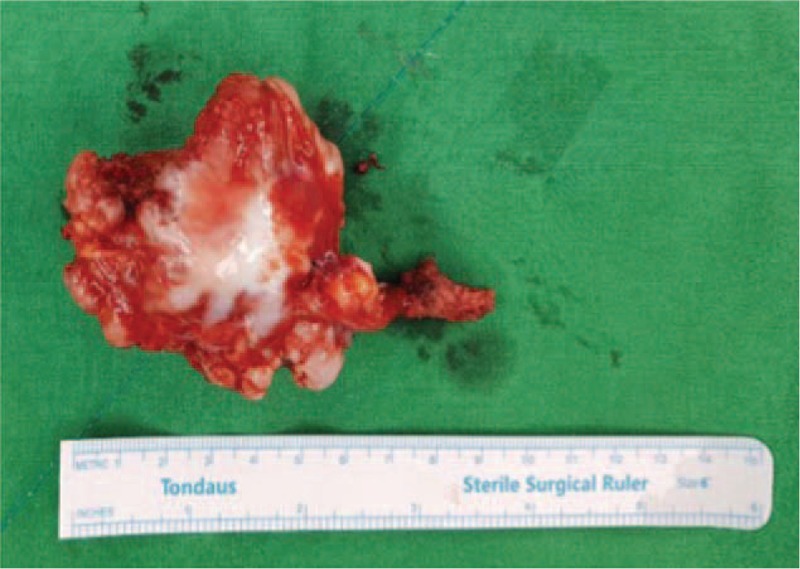
Excised specimen: It is a hard and exophytic mass composed of bone covered by a thick cartilage cap.

**Figure 4:**
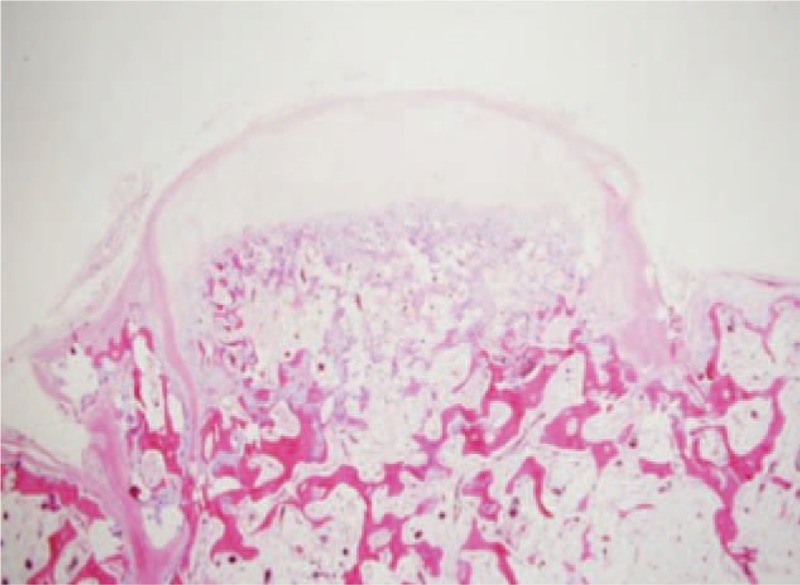
The specimen is a pedunculated bony protrusion from the parent bone (lower portion) with the cartilaginous cap. (H&E stain, ×12.5).

Surgery was performed under general anesthesia with the patient in lateral position. The shoulder was rotated internally, thereby lifting the medial border of the scapula away from the thoracic cage. The incision was made through the para-scapular approach. A muscle splitting approach of the trapezius and rhomboid was used to reach the bone. The pedunculated mass which was about 6×6×4 cm in dimension was located on the ventral aspect of the scapula, and the left rib and the chest wall were compressed. The mass was entirely excised. Grossly, the mass was hard and exophytic and composed of bone covered by a thick cartilage cap (Fig. 3). At the lower power view, it showed a pedunculated bony protrusion covered by a cartilaginous cap, consistent with osteochondroma (Fig. 4).

**Figure 5:**
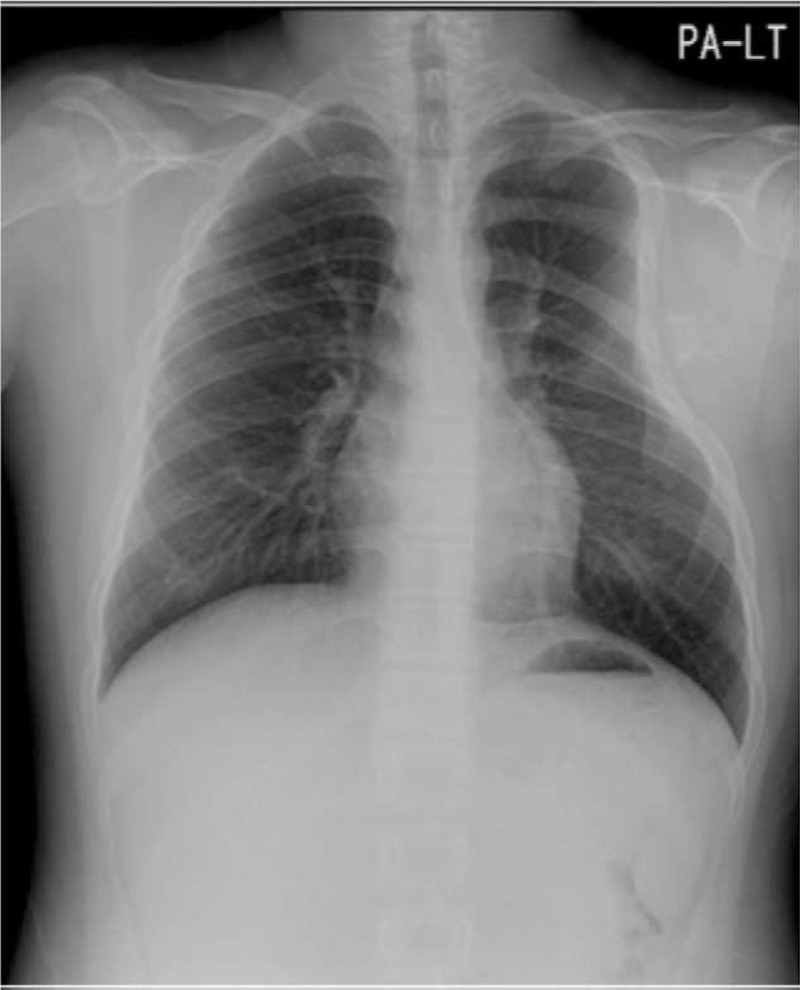
A 1-year follow-up chest anteroposterior radiographs shows no recurrence of osteochondroma and improvement of compression of chest wall.

The arm was immobilized by putting it in an abductor pillow brace for 3 weeks, and during that period pendulum exercise was permitted. The patient's symptoms resolved in the immediate postoperative period. At 1 year's follow-up, the patient was symptom free and there was no evidence of recurrence of the tumor (Fig. 5).

## 3 Discussion

Osteochondroma is the most common benign bone tumor.^[1]^ It is commonly seen in young patients, usually in those who are below 30 years of age, with a male: female ratio of > 1.5: 1.^[2]^ The long bones of the lower limbs especially around the knee are most commonly affected. After around the knee, the proximal portions of the femur and the humerus are the sites preferentially affected.^[2]^ On the other side, the involvement of the flat bones has been relatively rarely reported, and especially scapular lesions account for 4% of all described osteochondroma.^[6]^

There are very few reports about the involvement of ventral surface of the scapula. Solitary osteochondroma of the ventral side of scapula may produce static winging and snapping shoulder syndrome. The snapping scapula syndrome was first described by Boinet in 1867. The syndrome presents with pain in back and around the shoulder girdle with audible and/or palpable crepitus of scapula on scapulothoracic movement.^[6]^ Sometimes, there may be a large bursa formation associated with the mass. Furthermore, it can also produce various manifestations due to mass effect. A previous case report showed erosion of the ribs on the same side of ventral osteochondroma of the scapula.^[2]^ However, a study involving the mass effect and compression of the rib cage, even though produce chest pain as presented in our case report is extremely scarce.

Plain radiography is sufficient to diagnose the typical side of osteochondroma such as around the knee area. However, in certain bones such pelvis and the scapula, the plain radiography could be a limitation of diagnosis. A CT scan is useful to diagnose these bones.^[1]^ Although our case was well observed on x-ray because of the large size of the tumor, CT scan was very helpful to observe the overall margin clearly.

The thickness of the cartilaginous cap seen in the biopsy specimen is one of the predicting factors for malignant transformation. A cartilaginous cap with thickness <1 cm indicates a benign condition whereas a cap thicker than 2 cm should raise concern for malignant transformation.^[2,4]^ In our case, the cartilaginous cap thickness measured 2 to 3 mm. There were no clinical and radiological signs of recurrence of the illness in our patient at 1-year follow-up. Since osteochondroma usually stops growing at the time of closure of the physis, we will follow-up carefully until after closure of the physis.

## 4 Conclusion

Osteochondroma of the ventral scapula is very rare and complaint of chest pain is an atypical symptom. We recommend that in case of patients who have a history of osteochondroma and complaint of chest pain, surgeons should become suspicious of the presence of osteochondroma of the ventral scapula. In this situation, we recommend chest radiography, pulmonary function test, and chest CT for early detection and treatment.

## Acknowledgments

The authors would like to thank Soonchunhyang University Research Fund for the support.

## Author contributions

**Conceptualization:** Dong-il Chun, Jae-ho Cho, Jun Yong Kim, Sung Hun Won.

**Writing – review & editing:** Dong-il Chun, Sung Hun Won.

**Investigation:** Jae-ho Cho, In Ho Choi.

**Writing – original draft:** Young Yi, Jun Yong Kim, Sung Hun Won.

**Data curation:** Jae Heon Kim.

**Formal analysis:** Jae Heon Kim.
